# Outcome of Stage IV Completely Necrotic Wilms Tumour and Local Stage III Treated According to the SIOP 2001 Protocol

**DOI:** 10.3390/cancers13050976

**Published:** 2021-02-26

**Authors:** Raquel Dávila Fajardo, Rhoikos Furtwängler, Martine van Grotel, Harm van Tinteren, Claudia Pasqualini, Kathy Pritchard-Jones, Reem Al-Saadi, Beatriz de Camargo, Gema L. Ramírez Villar, Norbert Graf, Xavier Muracciole, Patrick Melchior, Daniel Saunders, Christian Rübe, Marry M. van den Heuvel-Eibrink, Geert O. Janssens, Arnauld C. Verschuur

**Affiliations:** 1 Department of Radiation Oncology, University Medical Center Utrecht, 3584 CX Utrecht, The Netherlands; G.O.R.Janssens@umcutrecht.nl; 2 Princess Máxima Center for Pediatric Oncology, 3584 CS Utrecht, The Netherlands; M.vanGrotel@prinsesmaximacentrum.nl (M.v.G.); m.m.vandenheuvel-eibrink@prinsesmaximacentrum.nl (M.M.v.d.H.-E.); 3 Department of Paediatric Oncology and Haematology, University Hospital of Saarland, 66421 Homburg, Germany; Rhoikos.Furtwaengler@uks.eu (R.F.); Norbert.Graf@uks.eu (N.G.); 4 Trial and Data Center, Princess Maxima Center for Pediatric Oncology, 3584 CS Utrecht, The Netherlands; h.vantinteren@prinsesmaximacentrum.nl; 5 Department of Paediatric Oncology, Institute Gustave Roussy, CEDEX, 94805 Villejuif, France; claudia.pasqualini@gustaveroussy.fr; 6 Developmental Biology & Cancer Research & Teaching Department, UCL Great Ormond Street Institute of Child Health, London WC1N 1EH, UK; k.pritchard-jones@ucl.ac.uk (K.P.-J.); reem.al-saadi@ucl.ac.uk (R.A.-S.); 7 Research Center, Brazilian National Cancer Institute, Rio de Janeiro 20230-240, Brazil; bdecamar@terra.com.br; 8 Department of Paediatric Oncology, Hospital Universitario Virgen del Rocío, 41013 Seville, Spain; glramirezv@gmail.com; 9 Department of Radiation Oncology, Assistance Publique Hôpitaux de Marseille, 13005 Marseille, France; Xavier.MURACCIOLE@ap-hm.fr; 10 Department of Radiation Oncology, University Hospital of Saarland, 66421 Homburg, Germany; Patrick.Melchior@uks.eu (P.M.); Christian.Ruebe@uks.eu (C.R.); 11 The Christie NHS Foundation Trust, Manchester M20 4BX, UK; Daniel.Saunders@christie.nhs.uk; 12 Department of Paediatric Oncology, La Timone Children’s Hospital, Assistance Publique Hôpitaux de Marseille, 13005 Marseille, France; Arnauld.VERSCHUUR@ap-hm.fr

**Keywords:** Wilms tumour, nephroblastoma, completely necrotic, metastatic disease

## Abstract

**Simple Summary:**

Around 15–20% of all Wilms tumour (WT) patients present with metastatic disease. Approximately 10% of these patients achieve complete necrosis after preoperative chemotherapy, which is associated with a favourable prognosis. The aim of this observational study is to describe the outcome of metastatic patients with completely necrotic (low-risk histology), local stage III WT treated according to the SIOP 2001 protocol, whether or not postoperative radiotherapy was applied.

**Abstract:**

Objective: Wilms tumour (WT) patients with a localised completely necrotic nephroblastoma after preoperative chemotherapy are a favourable outcome group. Since the introduction of the SIOP 2001 protocol, the SIOP– Renal Tumour Study Group (SIOP–RTSG) has omitted radiotherapy for such patients with low-risk, local stage III in an attempt to reduce treatment burden. However, for metastatic patients with local stage III, completely necrotic WT, the recommendations led to ambiguous use. The purpose of this descriptive study is to demonstrate the outcomes of patients with metastatic, completely necrotic and local stage III WT in relation to the application of radiotherapy or not. Methods and materials: all metastatic patients with local stage III, completely necrotic WT after 6 weeks of preoperative chemotherapy who were registered in the SIOP 2001 study were included in this analysis. The pattern of recurrence according to the usage of radiation treatment and 5 year event-free survival (EFS) and overall survival (OS) was analysed. Results: seven hundred and three metastatic WT patients were registered in the SIOP 2001 database. Of them, 47 patients had a completely necrotic, local stage III WT: 45 lung metastases (11 combined localisations), 1 liver/peritoneal, and 1 tumour thrombus in the renal vein and the inferior vena cava with bilateral pulmonary arterial embolism. Abdominal radiotherapy was administered in 29 patients (62%; 29 flank/abdominal irradiation and 9 combined with lung irradiation). Eighteen patients did not receive radiotherapy. Median follow-up was 6.6 years (range 1–151 months). Two of the 47 patients (4%) developed disease recurrence in the lung (one combined with abdominal relapse) and eventually died of the disease. Both patients had received abdominal radiotherapy, one of them combined with lung irradiation. Five-year EFS and OS were 95% and 95%, respectively. Conclusions: the outcome of patients with stage IV, local stage III, completely necrotic Wilms tumours is excellent. Our results suggest that abdominal irradiation in this patient category may not be of added value in first-line treatment, consistent with the current recommendation in the SIOP–RTSG 2016 UMBRELLA protocol.

## 1. Introduction

The Wilms tumour (WT), or nephroblastoma, is the most frequent paediatric renal tumour, which accounts for 80–90% of all tumours of the kidney in childhood [1]. Around 15–20% of all WT patients present with stage IV disease. The most frequent metastatic site is the lung, followed by liver, extra-abdominal lymph-node metastasis, and, relatively infrequently, bone or brain metastasis [2,3,4,5]. The usual treatment approach for WT combines the use of chemotherapy and surgery with the addition of radiotherapy based on stage and histology risk group. Over the past decades, the focus on improving risk stratification adapted treatment has resulted in an increased overall survival (OS) for patients with WT. At present, long-term OS exceeds 90% in localised disease and 80% in metastatic patients [4,6,7,8,9]. Achieving completely necrotic WT histology after preoperative chemotherapy is prognostically favourable. In the SIOP 9 study, the OS rate of patients with completely necrotic stage IV disease was 100% [10,11]. Furthermore, patients undergoing resection of lung metastases show high survival rates if no vital tumour cells are found in the specimen [4,8]. Since the introduction of the SIOP 2001 protocol, this excellent outcome has resulted in the omission of abdominal radiotherapy in patients with localised disease, completely necrotic stage III. Whether radiotherapy to the primary tumour area or to the metastatic sites is required in the case of patients with metastatic disease and completely necrotic, local stage III WT has never been assessed.

This report describes the outcomes of metastatic patients with completely necrotic (low-risk (LR) histology, as defined in the revised SIOP working classification of renal tumours of childhood), local stage III WT, treated according to the SIOP 2001 protocol, based on the use or non-use of postoperative radiotherapy (Tables S1 and S2).

## 2. Materials and Methods

### 2.1. Treatment Protocol

According to the SIOP 2001 protocol, all newly diagnosed patients with a metastatic intrarenal tumour received 6 weeks of preoperative chemotherapy (weekly intravenous (i.v.) vincristine (1.5 mg/m^2^) combined with actinomycin D (45 µg/kg) (every 2 weeks), and doxorubicin (50 mg/m^2^; weeks 1 and 5) (VAD)), followed by tumour nephrectomy and standard lymph node sampling [4,12]. Reference pathology assessment was performed in all cases. Postoperative treatment was determined by the local stage of the abdominal tumour, its histologic subtype, and the result of the radiological re-evaluation of the metastatic site at the time of surgery.

Patients with metastatic WT, local stage III and LR or intermediate-risk (IR) histology received postoperative VAD chemotherapy for 27 weeks (weekly i.v. vincristine combined with actinomycin D every 3 weeks and doxorubicin every 6 weeks, total cumulative dose not exceeding 300 mg/m^2^) if the metastatic lesions were absent or completely resected at time of nephrectomy, and no radiotherapy to the metastatic site was applied. In the presence of multiple, non-resectable, or incompletely resected metastasis, postoperative treatment consisted of four drugs: CDCV chemotherapy (high-risk regimen) continued for 34 weeks with etoposide (150 mg/m^2^) and carboplatin (200 mg/m^2^) for three consecutive days in weeks 4, 10, 13, 16, 22, 25, 28, and 34 (24 doses in total) combined with cyclophosphamide (450 mg/m^2^) for three consecutive days in weeks 1, 7, 19, and 31 (12 doses in total) and doxorubicin (50 mg/m^2^, total cumulative dose not exceeding 300 mg/m^2^) for one day only in weeks 1, 7, 19, and 31 (4 doses in total) (Figure S1). Flank/abdominal irradiation was indicated for local stage III IR, but not for LR histology (14.4 Gy in 8 fractions of 1.8 Gy, +/− a boost of 10.8 Gy in 6 fractions of 1.8 Gy to areas of macroscopic tumour rest). Radiotherapy to the metastases was indicated if persistent at re-evaluation in week 9 (15 Gy in 10 fractions of 1.5 Gy to both lungs, with optional boost of 10–15 Gy in 1.5 Gy per fraction to areas of gross residual disease after surgery; Figures S1 and S2). For LR stage IV patients, VAD postoperative chemotherapy could be considered as alternative to the four-drug regimen according to the decision of the local multidisciplinary tumour board. Abdominal radiotherapy was started at week 2 to 4 of postoperative chemotherapy for patients with metastatic complete remission at the time of tumour nephrectomy. If not, radiotherapy could be delayed until week 10 in an attempt to avoid overlap between the lung and abdominal fields. No specific recommendations regarding radiotherapy to the persistent metastases were made in SIOP 2001 for metastatic patients with completely necrotic, local stage III WT.

### 2.2. Statistical Analysis

Event-free survival and overall survival were calculated from the date of diagnosis. Event-free survival was considered as time to loco-regional or distant recurrence, or death from any cause. Overall survival was time to death from any cause. Event-free patients at the end of follow-up were censored at that moment. For the current study, 5 year event-free survival (EFS) and overall survival (OS) were calculated using the Kaplan–Meier method.

The median follow-up was calculated using the reverse Kaplan–Meier method. Statistical analysis was performed using the statistical software SAS version 9.2 and R version 4.01 [13].

## 3. Results

### 3.1. Patient Characteristics 

Between June 2001 and December 2017, 703 patients with metastatic WT were included in the SIOP 2001 study. Of these, 47 (7%) patients had histologically confirmed completely necrotic, local stage III WT. Twenty-one were males and 26 were females. Median age at diagnosis was 50 months (interquartile range 14–144). Median follow-up was 6.6 years (range 1–151 months). Forty-five had lung metastases (11 of them combined with other localisations), one patient had liver/peritoneal metastasis only, and one patient had a tumour thrombus in the renal vein and the inferior vena cava with bilateral pulmonary arterial embolism only. Since this last patient cannot strictly be considered as metastatic, in the absence of intraparenchymal lung metastasis, she was excluded from the statistical analysis, but she still is in continuous complete remission 12 years after initial diagnosis. In all 47 cases, information on radiotherapy was available. Abdominal radiotherapy was administered in 29 patients (62%; 29 flank/abdominal irradiation and 9 combined with lung irradiation). Eighteen patients received neither radiotherapy to the abdomen nor to the metastatic site. All 18 non-irradiated patients had lung metastases, either alone or combined with other localisations, and their lung metastatic status at the time of nephrectomy was as follows: 10 patients had shown complete response (CR) after chemotherapy alone, 2 patients had CR after surgery to the metastases, 5 patients had partial response after chemotherapy and incomplete resection or had multiple irresectable metastases, and in 1 patient, this information was missing. Patient, tumour, and treatment characteristics are depicted in Table 1.

### 3.2. Disease Control and Survival 

**Figure 1:**
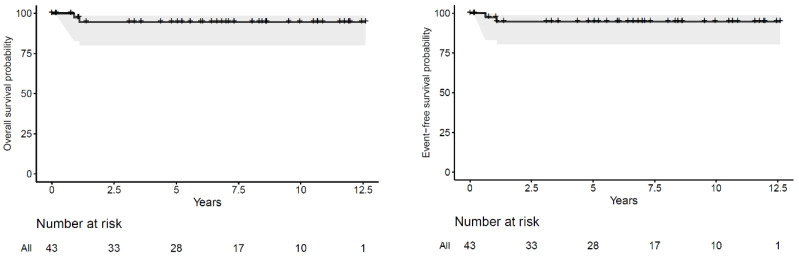
Overall survival (OS) and event-free survival (EFS) for patients with completely necrotic, local stage III, stage IV Wilms tumour.

Two of the 47 patients (4%) relapsed. Both developed lung recurrence, one of them (who had lung and liver metastasis at diagnosis) combined with concurrent abdominal relapse. Both patients eventually died from disease within the first 2 years post-diagnosis. Both patients had received abdominal radiotherapy during first-line treatment, combined with lung irradiation in one of them. No relapse occurred in patients treated without radiotherapy (Figure S3). Of the 47 patients, 18 achieved complete remission with chemotherapy alone at week six; only one of them received radiotherapy to the metastatic site. Among the 29 patients who did not attain complete remission at week six, 13 switched to the high-risk CDCV postoperative chemotherapy regimen. One of these patients relapsed. Details on relapse patterns are depicted in Table 2. The 5year EFS and OS were 95% (95% CI 88–100) and 95% (95% CI 88–100), respectively (Figure 1).

## 4. Discussion

In SIOP 2001, no specific recommendations on postoperative radiotherapy were made for patients with metastatic, completely necrotic, local stage III WT, achieved after 6 weeks of preoperative chemotherapy. Omission of radiotherapy was carried out in a significant number of patients. The results of the current analysis suggest that withdrawing radiotherapy from the treatment strategy in this category of patients does not impact either loco-regional control or survival.

Around 7–10% of WT patients demonstrate completely necrotic histology after preoperative chemotherapy [10,11]. As defined in the revised SIOP working classification of renal tumours of childhood, a completely necrotic condition is assessed if no viable tumour tissue is identified on gross and microscopic examination [14,15]. This histology has been correlated with good prognosis, reaching survival rates of 100% in patients with metastatic disease [4,11]. Over the past few decades, substantial treatment advances for WT have permitted a stepwise refinement of risk-adapted strategies in an attempt to reduce the treatment-related morbidity of WT survivors. In localised patients, the omission of doxorubicin in non-high-risk histology after preoperative chemotherapy has become current practice according to the SIOP–RTSG [16]. Similarly, the avoidance of flank/abdominal radiotherapy in local stage I–II non-anaplastic histology and of lung radiotherapy in the case of complete remission of lung metastasis (subsequent to either preoperative chemotherapy or to pulmonary metastasectomy) has been standardised [4,6,17,18]. Whether radiotherapy to the primary or metastatic site is required in metastatic patients with completely necrotic (LR), local stage III WT, is still a subject of discussion. In SIOP 93-01, patients with metastatic LR, local stage III WT, achieved after 6 weeks of preoperative chemotherapy consisting of actinomycin D (15 µgr/kg), vincristine (1.5 mg/m^2^) and epirubicin (50 mg/m^2^), received abdominal radiotherapy (15 Gy to the initial tumour volume with or without a boost of 10–15 Gy to areas of potential risk), and radiotherapy to the metastases if no complete response was attained at the time of re-evaluation after preoperative chemotherapy (15 Gy followed by optional boost of 10 Gy to areas of residual disease).

Based on the good outcomes of patients with completely necrotic WT, all stages combined, treated according to SIOP 9 (i.e., 5 year OS 98%), no abdominal radiotherapy was recommended for patients with a completely necrotic localised disease (stages I–III) in the SIOP 2001 protocol (Table S3) [11]. Since no clear recommendations were made on the indication of radiotherapy for patients with metastatic completely necrotic, local stage III WT, differences in interpretation of the protocol recommendations led to the appearance of two groups, where 40% of these patients did not receive radiotherapy and none of them developed recurrence. This finding suggests that further radiotherapy dose de-escalation in this patient category is safe, as was proven in the sequential SIOP studies for other risk categories depending on stage, histology, and response to preoperative chemotherapy [18,19,20,21]. In the current analysis, 44% of the patients who did not reach complete remission of the metastatic site after preoperative chemotherapy were treated postoperatively according to the high-risk chemotherapy regimen, showing no differences in outcomes in comparison to the group that received the three-drug combination regimen. This observation requires us to question the necessity of switching to high-risk chemotherapy, since the outcome seems similar to that of patients treated with VAD. The decision to switch to high-risk chemotherapy should be on a per patient basis, taking into consideration the decrease in metastatic burden (very good partial response) and preferably the histology of the metastases. Moreover, it should be emphasised that the only two relapses occurred in patients having received radiotherapy (one of them including pulmonary radiotherapy), while none of the 18 patients who did not receive any radiotherapy relapsed. However, the retrospective character of the study and the small sample size should be noted as limitations. In the absence of a feasible randomised clinical trial that can address this question, the excellent outcomes presented are encouraging. The strengths of this study are the consecutive prospective registration and data collection of all patients included in the SIOP 2001 study protocol.

Long-term survivors of Wilms tumours are at an increased risk of developing multifactorial treatment-related morbidity and mortality. The most frequent complications after abdominal radiotherapy are cardiovascular, followed by musculoskeletal development impairment and treatment-induced secondary malignancies, as well as metabolic, renal, and gonadal problems [22,23,24,25,26,27]. The avoidance of pulmonary radiotherapy may preclude medium- and long-term cardiac and respiratory morbidity in WT survivors. In addition, various respiratory disorders, such as reduced lung total capacity, interstitial pneumonia, and, consequently, exercise-induced dyspnoea, are common sequelae after pulmonary radiotherapy [28,29,30,31,32,33]. Radiotherapy alone may cause congestive heart failure, and the risk increases exponentially when combined with anthracyclines [25,34]. Musculoskeletal and soft tissue growth abnormalities, such as breast hypoplasia, hypothyroidism, and an increased risk of secondary malignancies, may also be induced by the use of pulmonary radiotherapy [29,30]. It is to be expected that the omission of abdominal and, if applicable, pulmonary radiotherapy in metastatic completely necrotic, local stage III WT patients will contribute to a risk reduction of treatment-related sequelae [8]. Therefore, the ongoing SIOP–RTSG UMBRELLA protocol does not recommend radiotherapy in these patients [1].

## 5. Conclusions

The results of this descriptive study demonstrate that the outcome of patients with stage IV, local stage III, completely necrotic Wilms tumours is excellent, and that the omission of radiotherapy, after preoperative chemotherapy, in first-line treatmentdoes not seem to have an impact on survival. Avoidance of radiotherapy in this patient category has the potential to reduce the treatment toxicity burden in Wilms tumour survivors.

## Figures and Tables

**Table 1:** Patient, tumour, and treatment characteristics of totally necrotic, local stage III, stage IV Wilms tumours.

		RT			No RT
		N = 29			N = 18
		Flank/Abdomen	Flank/Abdomen/Lung	Total	
		N = 20	N = 9	N = 29	
Gender	Male	9	2	11	10
	Female	11	7	18	8
Tumour site	Right	10	7	17	9
	Left	10	2	12	9
Reason for local stage III	SM positive	7	2	9	7
	LN positive	6	4	10	9
	SM and LN positive	4	3	7	0
	Tumour rupture	1	0	1	1
	Peritoneal implants	1	0	1	0
	SM positive and peritoneal implants	1	0	1	0
	NA	0	0	0	1
Metastatic site	Lung only	15	5	20	14
	Lung combined	3	4	7	4
	Liver/abdomen	1	0	1	0
	Other	1	0	1	0
Postoperative chemotherapy regimen	AVD	12	3	15	16
	AV-2	1	0	1	0
	High-risk	6	6	12	2
	NA	1	0	1	0
Metastatic status after preoperative chemotherapy and surgery	Metastases absent with chemotherapy alone	9	1	10	10
	Completely excised	3	2	5	2
	Incompletely excised or multiple irresectable	7	6	13	5
	NA	1	0	1	1
Recurrence status	Yes	1	1	2	0
	No	19	8	27	18
Collaborative group	GCBTTW	0	0	0	1
	GPOH	3	0	3	9
	SFCE	4	0	4	3
	SIOP-NL	9	2	11	4
	CCLG	4	7	11	1

Abbreviations: SM: surgical margin, LN: lymph node, UK: unknown, RT: radiotherapy, A: actinomycin D, V: vincristine, D: doxorubicin, NA: not available, GCBTTW: Brasil, GPOH: Germany and Austria, SFCE: France, SIOP-NL: all other European countries that registered through the SIOP-Office, The Netherlands, CCLG: United Kingdom.

**Table 2:** Patient and treatment summary.

Patient No	Age (Months)	Gender	Reason for Stage III	Metastasis CR at Week 6	Metastasis CR at Week 10 (After CHT and Surgery)	Metastasis Surgically Removed	Postoperative CHT Schema	Abdominal RT Directed	RT Dose (Gy) Elective/Boost	RT Metastasis Directed	Outcome
1	61	F	SM and LN +	Yes	Yes	No	AVD	Yes	14.4/10.8	No	CR/Alive
2	37	F	SM +	No	No	No	AVD	Yes	14.4	No	CR/Alive
3	26	F	SM +	Yes	Yes	No	AV-2	Yes	14.4	No	CR/Alive
4	87	M	SM and LN +	No	Yes	Yes	AVD	Yes	16	No	CR/Alive
5	37	M	SM and LN +	Yes	Yes	No	AVD	Yes	14.4/10.8	No	CR/Alive
6	50	F	LN +	No	No	No	High-risk	Yes	14.4/10.8	No	CR/Alive
7	43	M	SM +	No	No	No	High-risk	Yes	14.4	No	CR/Alive
8	70	F	LN +	Yes	Yes	No	AVD	Yes	15	No	CR/Alive
9	77	M	LN +	Yes	Yes	No	High-risk	Yes	14.4/10.8	Yes	CR/Alive
10	28	M	LN +	No	No	No	AVD	Yes	14.4	No	CR/Alive
11	39	M	Rupture	Yes	Yes	No	AVD	Yes	14.4	No	CR/Alive
12	68	M	Peritoneal implants	No	Yes	Yes	AVD	Yes	21	No	CR/Alive
13	53	M	SM +	Yes	Yes	No	AVD	Yes	14.4	No	CR/Alive
14	28	F	SM +	No	No	No	High-risk	Yes	14.4	No	CR/Alive
15	36	M	Peritoneal implants and SM +	No	No	Yes	High-risk	Yes	19.5	No	CR/Alive
16	85	M	SM +	No	Yes	Yes	High-risk	Yes	20	No	CR/Alive
17	48	F	LN +	No	No	No	High-risk	Yes	14.4	No	CR/Alive
18	69	F	LN +	No	No	No	AVD	Yes	14.4	Yes	CR/Alive
19	44	M	SM and LN +	No	Yes	Yes	High-risk	Yes	15	Yes	CR/Alive
20	95	F	SM and LN +	No	No	No	AVD	Yes	14.4	Yes	CR/Alive
21	14	F	LN +	No	No	No	High-risk	Yes	15	Yes	CR/Alive
22	49	F	SM and LN +	Yes	Yes	No	AVD	Yes	14.4	No	CR/Alive
23	48	F	LN +	No	No	No	High-risk	Yes	14.4/7.5	Yes	Relapse/Dead
24	97	F	SM +	No	No	No	AVD	Yes	14.4/10.8	Yes	CR/Alive
25	41	F	LN +	Yes	Yes	No	AVD	Yes	14.4/10.8	No	CR/Alive
26	44	F	SM +	No	NA	NA	NA	Yes	21	No	Relapse/Dead
27	105	F	SM +	No	Yes	Yes	High-risk	Yes	15/6	Yes	CR/Alive
28	99	F	LN +	Yes	Yes	No	AVD	Yes	21	No	CR/Alive
29	97	F	SM and LN +	No	No	No	High-risk	Yes	14.4/10.8	Yes	CR/Alive
30	38	F	LN +	Yes	Yes	No	AVD	No	-	No	CR/Alive
31	48	F	SM +	Yes	Yes	No	AVD	No	-	No	CR/Alive
32	43	F	SM +	No	NA	NA	AVD	No	-	No	CR/Alive
33	75	M	LN +	Yes	Yes	No	AVD	No	-	No	CR/Alive
34	23	M	SM +	Yes	Yes	No	AVD	No	-	No	CR/Alive
35	49	M	LN +	No	No	No	High-risk	No	-	No	CR/Alive
36	87	M	LN +	No	No	No	AVD	No	-	No	CR/Alive
37	35	M	LN +	No	No	No	High-risk	No	-	No	CR/Alive
38	68	F	SM +	No	No	No	AVD	No	-	No	CR/Alive
39	67	M	LN +	Yes	Yes	No	AVD	No	-	No	CR/Alive
40	40	M	LN +	Yes	Yes	No	AVD	No	-	No	CR/Alive
41	144	M	LN +	No	Yes	Yes	AVD	No	-	No	CR/Alive
42	52	F	LN +	No	No	No	AVD	No	-	No	CR/Alive
43	75	M	LN +	Yes	Yes	No	AVD	No	-	No	CR/Alive
44	53	F	SM +	No	Yes	No	AVD	No	-	No	CR/Alive
45	72	M	SM +	Yes	Yes	No	AVD	No	-	No	CR/Alive
46	50	F	Rupture	No	No	No	AVD	No	-	No	CR/Alive
47	59	F	SM +	No	Yes	No	AVD	No	-	No	CR/Alive

Abbreviations: CR: complete remission, CHT: chemotherapy, RT: radiotherapy, F: female, M: male, NA: not available, SM +: surgical margins positive, LN +: lymph node(s) positive, A: actinomycin D, V: vincristine, D: doxorubicin.

## Data Availability

Data are contained within the article or Supplementary Materials.

## Supplementary Materials

The following are available online at https://www.mdpi.com/2072-6694/13/5/976/s1, Table S1: Revised SIOP working classification of renal tumours of childhood (2001), Table S2: SIOP staging criteria for renal tumours of childhood, Table S3: Summary of postoperative treatment for patients with localised disease Wilms tumour treated according to the SIOP 2001 protocol. Figure S1: Summary of postoperative treatment for stage IV, non-high-risk histology Wilms tumour (SIOP 2001 protocol), Figure S2: Summary of indications for postoperative radiotherapy and definition of clinical target volume as per SIOP 2001 protocol; Figure S3: Patient population flowchart.
