# Supplementary material for: Outcome of Stage IV Completely Necrotic Wilms Tumour and Local Stage III Treated According to the SIOP 2001 Protocol
Source: Cancers (Basel). 2021 Feb 26;13(5):976. doi: 10.3390/cancers13050976 (PMC7956604; doi:10.3390/cancers13050976)
Supplement: Supplementary file 1 [file cancers-13-00976-s001.pdf]

Outcome of Stage IV Completely Necrotic Wilms Tumour and Local Stage III Treated According to the SIOP 2001 Protocol

Raquel Dávila Fajardo, Rhoikos Furtwängler, Martine van Grotel, Harm van Tinteren, Claudia Pasqualini, Kathy Pritchard-Jones, Reem Al-Saadi, Beatriz de Camargo, Gema L. Ramírez Villar, Norbert Graf, Xavier Muracciole, Patrick Melchior, Daniel Saunders, Christian Rübe, Marry M. van den Heuvel-Eibrink, Geert O. Janssens, Arnauld C. Verschuur

Table S1. Revised SIOP working classification of renal tumours of childhood (2001) [15].

Risk Group	Pretreated Cases	Primary Nephrectomy Cases
Low-risk	Mesoblastic nephroma<br>Cystic partially differentiated nephroblastoma<br>Completely necrotic nephroblastoma	Mesoblastic nephroma<br>Cystic partially differentiated nephroblastoma
Intermediate-risk	Nephroblastoma-epithelial type<br>Nephroblastoma-stromal type<br>Nephroblastoma-mixed type<br>Nephroblastoma-regressive type<br>Nephroblastoma-focal anaplasia	Non-anaplastic nephroblastoma and its variants<br>Nephroblastoma-focal anaplasia
High-risk	Nephroblastoma-blastemal type<br>Nephroblastoma-diffuse anaplasia<br>Clear cell sarcoma of the kidney<br>Rhabdoid tumour of the kidney	NephroblastomaÐdiffuse anaplasia<br>Clear cell sarcoma of the kidney<br>Rhabdoid tumor of the kidney

Table S2. SIOP staging criteria for renal tumours of childhood [15].

Stage	Criteria
Stage I	a) The tumour is limited to kidney or surrounded with a fibrous pseudocapsule if outside of the normal contours of the kidney. The renal capsule or pseudocapsule may be infiltrated with the tumour but it does not reach the outer surface, and it is completely resected (resection margins 'clear')<br>b) The tumour may be protruding ('bulging') into the pelvic system and 'dipping' into the ureter (but it is not infiltrating their walls)<br>c) The vessels of the renal sinus are not involved<br>d) Intrarenal vessel involvement may be present
	Fine needle aspiration or percutaneous core needle biopsy ('tru-cut') does not upstage the tumour but the size of the needle gauge should be mentioned to the pathologist<br>*The presence of necrotic tumour or chemotherapy-induced change in the renal sinus and/or within the perirenal fat should not be regarded as a reason for upstaging a tumour providing it is completely excised and does not reach the resection margins*
Stage II	a) The tumour extends beyond kidney or penetrates through the renal capsule and/or fibrous pseudocapsule into peri-renal fat but is completely resected (resection margins 'clear')<br>b) Tumour infiltrates the renal sinus and/or invades blood and lymphatic vessels outside the renal parenchyma but it is completely resected

	c) Tumour infiltrates adjacent organs or vena cava but is completely resected
Stage III	a) Incomplete excision of the tumour which extends beyond resection margins (gross or microscopical tumour remains post-operatively)<br>b) Any abdominal lymph nodes are involved<br>c) Tumour rupture before or intra-operatively (irrespective of other criteria for staging)<br>d) The tumour has penetrated through the peritoneal surface<br>e) Tumour implants are found on the peritoneal surface<br>f) The tumour thrombi present at resection margins of vessels or ureter, transsected or removed piece-meal by surgeon<br>g) The tumour has been surgically biopsied (wedge biopsy) prior to preoperative chemotherapy or surgery.<br>*The presence of necrotic tumour or chemotherapy-induced changes in a lymph node or at the resection margins is regarded as proof of previous tumour with microscopic residue and therefore the tumour is assigned stage III (because a possibility that some viable tumour is left behind in the adjacent lymph node or beyond resection margins)*
Stage IV	Haematogeneous metastases (lung, liver, bone, brain, etc.) or lymph node metastases outside the abdomino-pelvic region
Stage V	Bilateral renal tumours at diagnosis. Each side should be substaged according to above classifications

Table S3. Summary of postoperative treatment for patients with localised disease Wilms tumour treated according to SIOP 2001 protocol. Abbreviations: A: actinomycin-D, V: vincristine, R: randomization, D/DOX: doxorubicin, RT: radiotherapy.

	STAGE I	STAGE II	STAGE III
LOW RISK	NO FURTHER TREATMENT	AV-2	AV-2
INTERMEDIATE RISK	AV-1	R< DOX + (AVD) / DOX – (AV-2)	R< RT/DOX + (AVD) / RT/DOX – (AV-2)
HIGH RISK	AVD	HIGH RISK + RT	HIGH RISK + RT

STAGE IV, POST-OPERATIVE TREATMENT

A. METASTASES ABSENT OR COMPLETELY RESECTED BY THE SURGEON
(See C for HIGH RISK PRIMARY TUMOUR)

<u>LOCAL STAGE I - II:</u> NO IRRADIATION

<u>LOCAL STAGE III:</u> ABDOMINAL IRRADIATION

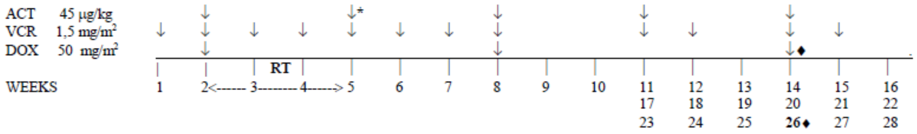

♦ **no DOX in week 26, so that the total dose does not exceed 300 mg/m²**

*** Reduce dose of ACT for 50% when RT is within 14 days of this administration (week 5).**

If body weight < 12 kg: dose reduction to 2/3 for each drug
Major intolerance: doses on the next course should be reduced to 2/3 (see section 8)

STAGE IV, POST-OPERATIVE TREATMENT

B. MULTIPLE INOPERABLE METASTASES OR INCOMPLETELY RESECTED

LOCAL STAGE I - II: NO ABDOMINAL IRRADIATION

LOCAL STAGE III: WITH ABDOMINAL IRRADIATION

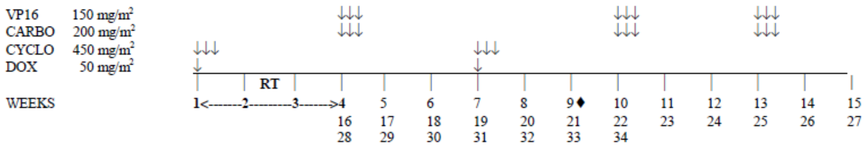

Pulmonary irradiation: ♦ evaluate and decide on pulmonary irradiation in week 9
Pneumocystis carinii pneumoniae prophylaxis in case of pulmonary irradiation

If body weight < 12 kg: dose reduction to 2/3 for each drug
Major intolerance: doses on the next course should be reduced to 2/3 (see section 8)

Figure S1. Summary of postoperative treatment for stage IV, non-high-risk histology Wilms tumour (SIOP 2001 protocol).

Indications for post-operative flank RT:

- Histologically intermediate risk, stage III (nodes positive N+, residual disease left after surgery, tumour rupture)

- High risk, stage II, except blastemal type

- High risk, stage III

- Stage IV and stage V according to local stage

Indications for post-operative whole abdominal RT:
Whole abdominal RT is indicated for DIFFUSE intra-abdominal tumour or GROSS pre-operative or peri-operative rupture.

Abdominal/flank RT will start as soon as possible within 2 weeks after abdominal surgery.
If there is an expectation of surgery for lung metastases or lung RT, abdominal RT will be postponed. In case of lung surgery abdominal RT will start after this surgery. In case of no lung surgery abdominal RT starts after week 9 whether or not in combination with lung RT.

Indications for pulmonary RT:
Residual tumour tissue in the lungs is visible on a chest X-ray or CT scan after the commencement of pre-operative chemotherapy, and that this residual tumour is not completely excised and if post-operative chemotherapy according to the high risk protocol does not lead to a complete remission, by week 9.
RT should not be given if a CR is achieved at week 9 of postoperative chemotherapy.
Complete remission: no abnormalities seen on chest X-ray or chest CT-scan.
RT is also indicated in case of a histological high risk primary tumour, regardless of metastatic response.

Indications for hepatic RT:
Liver metastases which do not respond completely to chemotherapy and which cannot be completely resected with negative margins.

Indications for RT to other metastatic sites:
Patients with haematogenous metastases to the brain (whole brain RT) and/or bone metastases (focal RT) at diagnosis, should be treated with the appropriate RT fields regardless of response to chemotherapy.

CLINICAL TARGET VOLUME (CTV)

Flank RT
CTV: This encompasses the extent of post-chemotherapy and pre-operative macroscopic tumour and the kidney according to the surgical and histopathological reports and according to the extent on CT-scan/ ultrasonography. The margin for CTV is 1 cm.

If there is no pre-operative CT-scan CTV is delineated by clips at the boundaries of the tumour and kidney placed by the surgeon during surgery. The margin for CTV is 1 cm beyond the clips.

The **treated volume** should extend across the midline to achieve homogeneous irradiation of the full width of the vertebral bodies.

Boosts for residual macroscopic disease
CTV: This should encompass the extent of macroscopic residual disease after surgery with a margin of 1 cm. If there is an indication for RT of the paraaortic lymphnodes the cranial field border should be at the thoracic vertebra T-10-TV-11 level while almost 50% of the celiac axis arises from the aorta at the level of the pedicle of the 12th vertebral body (2).
Again the full width of the vertebral bodies should receive a homogeneous dose.

Whole abdominal RT
CTV: This includes the entire abdominal contents and peritoneum extending from the dome of the diaphragm to the pelvic floor (lower border of obturator foramen).

Pulmonary RT
CTV: This encompasses both lungs including the apices and costo-diaphragmatic recesses. If abdominal radiotherapy also has to be given, both fields should be matched in order to avoid any gap or overlap.

Liver RT
CTV: This includes the extent of incompletely resected tumour with a margin of 2 cm.

RT for brain metastases
CTV: the whole brain is treated.

RT for haematogenous metastases to bone
CTV: For bone metastases it is not necessary to treat the entire bone. The field includes the obvious disease visible on imaging examination, with a margin of not less than 3 cm in any direction.

Figure S2. Summary of indications for postoperative radiotherapy and definition of clinical target volume as per SIOP 2001 protocol.

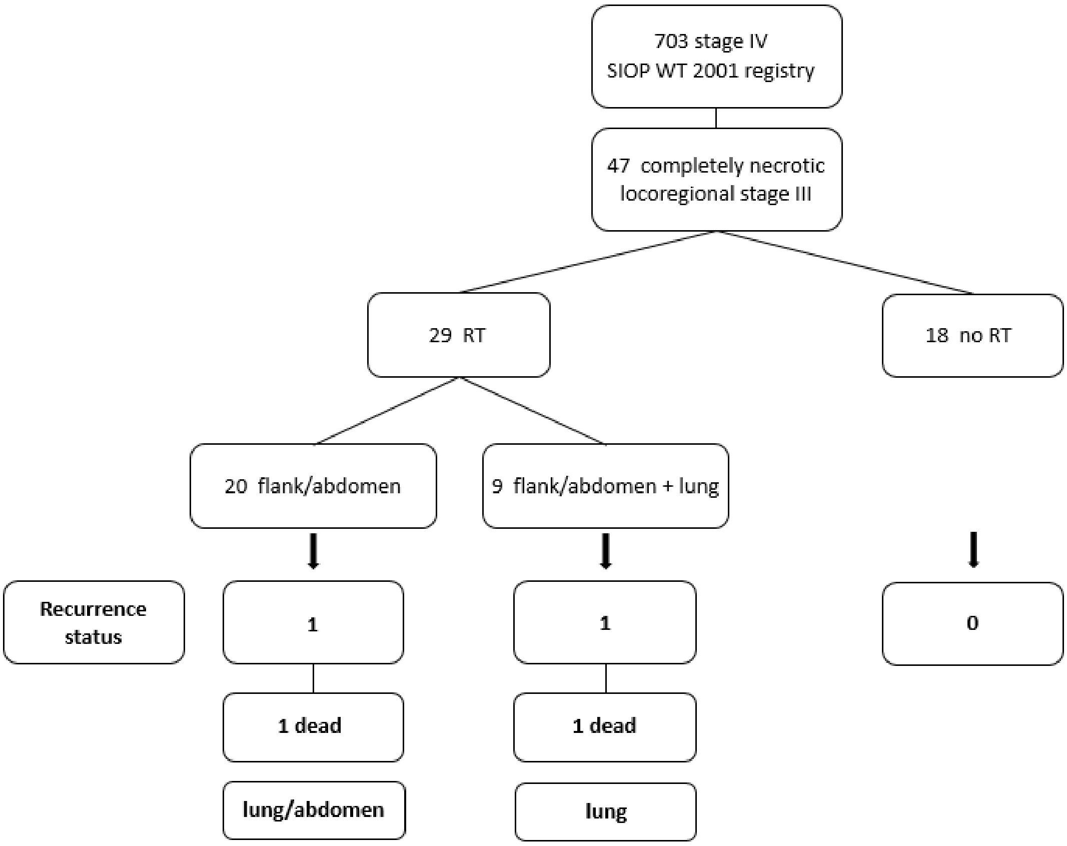

Figure S3. Patient population flow-chart. *Abbreviations:* WT: Wilms tumour, RT: radiotherapy.
